# The Design and Evaluation of an l-Dopa–Lazabemide Prodrug for the Treatment of Parkinson’s Disease

**DOI:** 10.3390/molecules22122076

**Published:** 2017-11-27

**Authors:** Monique Hoon, Jacobus P. Petzer, Francois Viljoen, Anél Petzer

**Affiliations:** 1 Pharmaceutical Chemistry, School of Pharmacy and Centre of Excellence for Pharmaceutical Sciences, North-West University, Private Bag X6001, Potchefstroom 2520, South Africa; 21087113@nwu.ac.za (M.H.); jacques.petzer@nwu.ac.za (J.P.P.); 2 Pharmacology, School of Pharmacy and Centre of Excellence for Pharmaceutical Sciences, North-West University, Private Bag X6001, Potchefstroom 2520, South Africa; francois.viljoen@nwu.ac.za

**Keywords:** lazabemide, l-dopa, prodrug, monoamine oxidase, MAO, inhibition, physicochemical properties

## Abstract

l-Dopa, the metabolic precursor of dopamine, is the treatment of choice for the symptomatic relief of the advanced stages of Parkinson’s disease. The oral bioavailability of l-dopa, however, is only about 10% to 30%, and less than 1% of the oral dose is estimated to reach the brain unchanged. l-Dopa’s physicochemical properties are responsible for its poor bioavailability, short half-life and the wide range of inter- and intrapatient variations of plasma levels. An l-dopa–lazabemide prodrug is proposed to overcome the problems associated with l-dopa absorption. Lazabemide is a monoamine oxidase (MAO)-B inhibitor, a class of compounds that slows the depletion of dopamine stores in Parkinson’s disease and elevates dopamine levels produced by exogenously administered l-dopa. l-Dopa was linked at the carboxylate with the primary aminyl functional group of lazabemide via an amide, a strategy which is anticipated to protect l-dopa against peripheral decarboxylation and possibly also enhance the membrane permeability of the prodrug. Selected physicochemical and biochemical properties of the prodrug were determined and included lipophilicity (logD), solubility, passive diffusion permeability, p*K*_a_, chemical and metabolic stability as well as cytotoxicity. Although oral and i.p. treatment of mice with the prodrug did not result in enhanced striatal dopamine levels, 3,4-dihydroxyphenylacetic acid (DOPAC) levels were significantly depressed compared to saline, l-dopa and carbidopa/l-dopa treatment. Based on the results, further preclinical evaluation of the l-dopa–lazabemide prodrug should be undertaken with the aim of discovering prodrugs that may be advanced to the clinical stages of development.

## 1. Introduction

**Figure 1:**
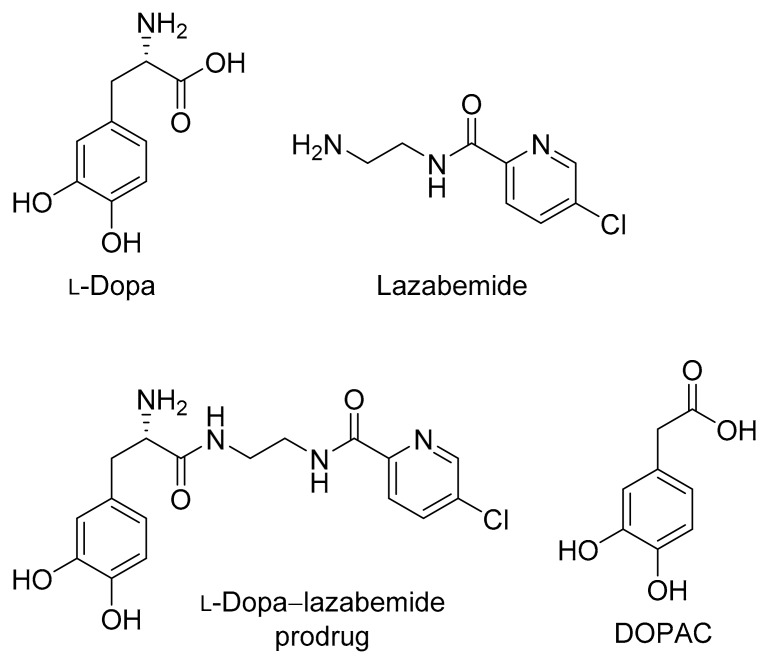
The structures of l-dopa, lazabemide and the l-dopa–lazabemide prodrug. The structure of DOPAC, a major metabolite of dopamine, is also shown.

Parkinson’s disease is a progressive, neurodegenerative disorder which is caused by the loss of dopaminergic neurons from the substantia nigra pars compacta in the brain [1]. The dopaminergic neurons which degenerate in Parkinson’s disease are specifically those of the nigrostriatal pathway that delivers dopamine to the striatum. The resulting functional deficit of dopamine in the striatum is responsible for the motor symptoms observed in Parkinson’s disease [1]. Ever since its early clinical use in the 1960s, l-dopa has remained the most effective treatment for Parkinson’s disease (Figure 1) [2,3]. l-Dopa is the direct metabolic precursor of dopamine and, in contrast to dopamine, permeates the blood–brain barrier by carrier-mediated transport [4]. l-Dopa may therefore be considered to be a prodrug of dopamine. Once in the brain, l-dopa can be converted to dopamine, thus effectively replacing the lost dopamine in the striatum [5]. Although l-dopa is absorbed from the gastrointestinal tract via the large neutral amino acid (LNAA) transport system, it is extensively decarboxylated in the gastrointestinal tract by the enzyme aromatic l-amino acid decarboxylase (AADC), and only approximately 30% of the l-dopa dose reaches the systemic circulation unchanged [2,6]. l-Dopa is further decarboxylated in the peripheral tissues, which further reduces the bioavailability to the brain. It is estimated that less than 1% of the administered oral dose of l-dopa reaches the brain unchanged [4]. To improve the bioavailability of l-dopa and to reduce peripheral dopaminergic side effects such as cardiac arrhythmias, hypotension, nausea and vomiting (due to the peripheral conversion of l-dopa to dopamine), l-dopa is combined with an AADC inhibitor such as carbidopa or benserazide [7]. When AACD is inhibited, 3-*O*-methylation catalysed by catechol-*O*-methyltransferase (COMT), however, becomes a dominant metabolic pathway for l-dopa, and the resulting metabolite, 3-*O*-methyldopa, accumulates in the peripheral and central tissues [8]. Several clinical observations have shown that poor response to l-dopa therapy is associated with high plasma levels of 3-*O*-methyldopa [9]. This is most likely because 3-*O*-methyldopa competes with l-dopa for transport across the blood–brain barrier. COMT inhibitors (e.g., entacapone) are thus also used as adjuncts to l-dopa in Parkinson’s disease [10,11].

Although oral l-dopa is a highly effective treatment for Parkinson’s disease, long-term use is associated with response fluctuations and involuntary movements, termed l-dopa-induced dyskinesias. These are, in part, due to irregular absorption (due to erratic gastric emptying), peripheral metabolism, a short half-life of approximately 90 min and limited transport across the blood–brain barrier [12,13]. In this respect, competition with dietary amino acids at transporters in the gastrointestinal tract and brain microvasculature may significantly affect l-dopa absorption and delivery to the brain [14,15]. Efforts have thus been made to develop new oral l-dopa formulations which may address some of the absorption, metabolism and delivery issues of l-dopa [2,3]. Several of these have entered clinical trials with IPX066, an l-dopa–carbidopa oral formulation combining immediate release (IR) and extended release (ER), having recently been approved [2]. Another approach to overcome the problems with peripheral l-dopa metabolism and delivery difficulties is to alter the physicochemical properties of l-dopa by designing prodrugs [4]. For example, XP21279 is a prodrug that is absorbed from the small and large intestines by high-capacity nutritional transporters [3]. After absorption, this compound is rapidly metabolised to l-dopa with a relative bioavailability of approximately 90% compared to l-dopa–carbidopa combination therapy [16,17].

**Figure 2:**
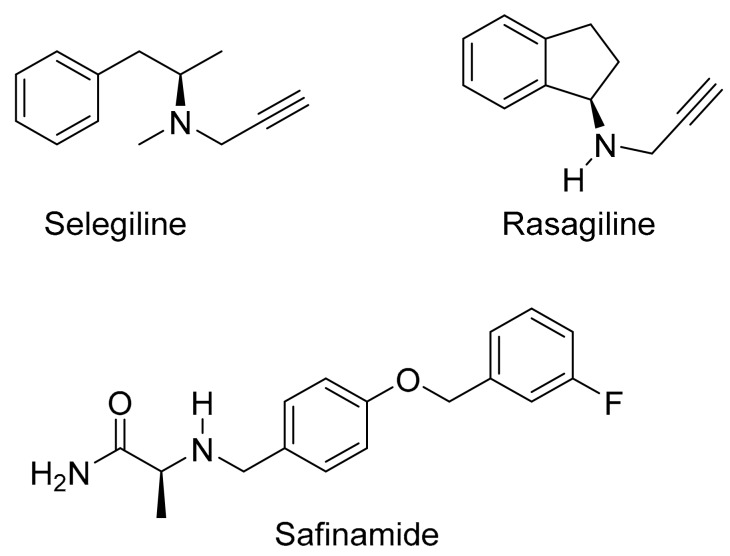
The structures of selegiline, rasagiline and safinamide.

The present study proposes a novel l-dopa–lazabemide prodrug to overcome the problems associated with l-dopa absorption and metabolism. Such a prodrug that links synergistic drugs is also known as a codrug or mutual prodrug. Lazabemide is a specific inhibitor of monoamine oxidase (MAO)-B, with a reversible mechanism-based mode of action (Figure 1) [18,19,20,21]. Lazabemide causes rapid and complete MAO-B inhibition with enzyme activity returning to baseline values by 36 h after drug discontinuation [22,23]. A dose of at least 0.4 mg/kg lazabemide given every 12 h provides >90% inhibition of brain MAO-B in patients with early Parkinson’s disease [23]. MAO-B inhibitors are considered useful agents in the therapy of Parkinson’s disease and are frequently combined with l-dopa [24]. By blocking the central MAO-B-catalysed metabolism of dopamine, these drugs are thought to slow the depletion of dopamine stores and to elevate dopamine levels produced by exogenously administered l-dopa [25]. In addition, MAO-B inhibitors may also protect against neurodegeneration in Parkinson’s disease, presumably by reducing the formation of injurious byproducts of the MAO-B catalytic cycle [26]. These byproducts, hydrogen peroxide and aldehyde species, may lead to neuronal death if not efficiently cleared from the central nervous system (CNS). Considering that MAO-B activity increases with age, the inhibition of this enzyme seems particularly relevant in Parkinson’s disease [27]. MAO-B inhibitors that are currently registered for the treatment of Parkinson’s disease are selegiline and rasagiline, irreversible mechanism-based inhibitors, and safinamide, a reversibly acting drug (Figure 2) [28,29]. Unfortunately, the development of lazabemide as an inhibitor of MAO-B for the treatment of Parkinson’s disease has been discontinued.

For the l-dopa–lazabemide prodrug, we have selected to link l-dopa at the carboxylate with the primary aminyl functional group of lazabemide via an amide (Figure 1). This would protect the carboxylic acid of l-dopa against peripheral decarboxylation and, since lazabemide is amphiphilic and associates with the phospholipid bilayer of membranes [30], the prodrug may exhibit enhanced membrane permeability compared to l-dopa. With the presence of the ionisable aminyl functional group, the catechol hydroxyl groups as well as the lipophilic chloropyridinyl and catechol phenyl moieties, it is expected that the amphiphilic nature of lazabemide will be retained in the prodrug. Amphiphilic compounds possess both hydrophilic and lipophilic properties, which are required for aqueous solubility and membrane permeability by passive diffusion, the two most important determinants of absorption from the gastrointestinal tract. In this respect, the catechol hydroxyl and ionisable amine groups present in the prodrug represent the hydrophilic nature while the chloropyridine and catechol phenyl moieties provide hydrophobicity. Although amides, in general, do not hydrolyse readily, amides of amino acids (e.g., l-dopa) are known to hydrolyse in vivo and the proposed prodrug is expected to be activated after administration [31]. The increased stability of the amide link compared to the more traditional ester between a drug and its carrier could possibly allow the prodrug more time to diffuse into the brain prior to this hydrolysis event. Furthermore, unlike l-dopa, the prodrug does not contain the acidic carboxylate group. Acidic moieties are known to reduce membrane permeation of small organic compounds [32]. With an enhanced lipophilicity, the prodrug may be absorbed from the gastrointestinal tract by passive diffusion, thus leading to improved bioavailability compared to l-dopa [32]. It is therefore anticipated that the prodrug will facilitate improved absorption and delivery of l-dopa to the brain and reduce peripheral decarboxylation. In addition, after activation of the prodrug, the released lazabemide may inhibit central MAO-B and thus enhance the levels of dopamine derived from l-dopa. Although the development of lazabemide has been discontinued due to liver toxicity, the l-dopa–lazabemide prodrug could serve as proof-of-concept for the feasibility of a prodrug that combines l-dopa with a MAO-B inhibitor [33].

## 2. Results

### 2.1. Synthesis of the l-Dopa–Lazabemide Prodrug

The synthesis of lazabemide has been reported [34]. Lazabemide was synthesised from 5-chloro-cyanopyridine and ethylenediamine.

**Figure 3:**
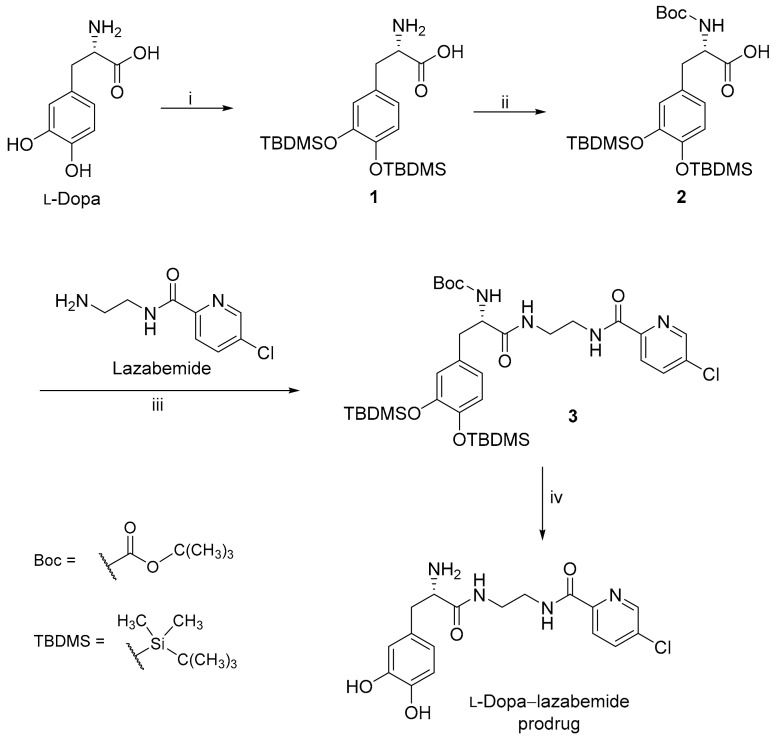
The protection of l-dopa and the synthesis of the l-dopa–lazabemide prodrug. Key: (i) r.t., 18 h, DBU, TBDMS-Cl; (ii) THF/H_2_O, NaHCO_3_, di-*tert*-butyl dicarbonate (Boc_2_O); (iii) BOP, NMM, CH_2_Cl_2_, 24 h; (iv) 4 M HCl (dioxane), 48 h, 55 °C.

Lazabemide was subsequently conjugated to the carboxylic acid functional group of l-dopa via an amide (Figure 3). A literature review showed that similar conjugation reactions have previously been carried out [35] and the carboxylic acid of l-dopa was, for example, conjugated to leucine, phenylalanine and valine. l-Dopa was firstly protected at the catechol OH groups using *tert*-butyldimethylsilyl chloride (TBDMS-Cl) in acetonitrile. Both phenolic positions on free l-dopa are selectively silylated by the reaction with TBDMS-Cl in the presence of 1,8-diazabicyclo[5.4.0]undec-7-ene (DBU). This step yields l-dopa protected on both phenolic positions, denoted as l-dopa(TBDMS)_2_ (**1**). The aminyl NH_2_ group of l-dopa was subsequently protected using di-*tert*-butyl dicarbonate (Boc_2_O) in THF. This introduces the *tert*-butoxycarbonyl (*t*-Boc) group on the primary amine of l-dopa(TBDMS)_2_ yielding the Boc-l-dopa(TBDMS)_2_ (**2**).

In the next step, lazabemide was conjugated to the protected l-dopa, Boc-l-dopa(TBDMS)_2_ (**2**), in the presence of BOP [(1*H*-benzotriazol-1-yloxy)tris(dimethylamino)phosphonium hexafluorophosphate] with dichloromethane as solvent. *N*-Methylmorpholine (NMM) served as base in this reaction. This reaction yielded the protected l-dopa–lazabemide prodrug (**3**). The removal of the TBDMS and Boc protective groups from the protected l-dopa–lazabemide prodrug (**3**) was the last step of the reaction sequence. The protected l-dopa–lazabemide prodrug (**3**) was treated with a variety of deprotection agents of which complete deprotection was achieved with 95% trifluoroacetic acid (TFA) after 24 h. Another useful reagent was HCl (4 M in dioxane). After treatment of **3** with the HCl reagent for 48 h at 55 °C, deprotection to a level of 95% was achieved. It may thus be concluded that both TFA and HCl are appropriate for the deprotection of protected l-dopa dipeptides. The deprotection reaction yielded the final product, the l-dopa–lazabemide prodrug.

**Figure 4:**
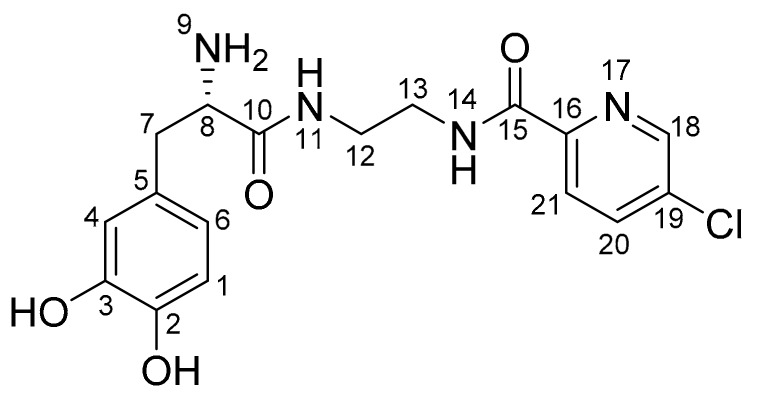
Atom numbering scheme for the l-dopa–lazabemide prodrug.

The structure of the l-dopa–lazabemide prodrug was characterised by ^1^H-NMR and ^13^C-NMR, as well as by mass spectroscopy (see Supplementary Materials for spectra). In both the ^1^H-NMR and ^13^C-NMR spectra, the appropriate signals were observed for the proposed prodrug. In the ^13^C-NMR spectrum, the amount of signals and their chemical shifts are in correspondence to what is expected for the proposed structure of the l-dopa–lazabemide prodrug. In the ^1^H-NMR spectrum, the amount of signals, their integration values, multiplicities and their chemical shifts are in correspondence to what is expected for the proposed structure of the l-dopa–lazabemide prodrug. In this respect, the CH_2_ protons of the l-dopa moiety (C7) correspond to the signals at 2.75–2.78 and 2.86–2.89 ppm (the signals integrate for 1 proton each), while the CH α-carbon (C8) corresponds to part of the multiplet at 3.30–3.36 ppm (the signal integrates for three protons) (Figure 4). The aromatic l-dopa protons (on C1, C4 and C6) correspond to the doublet at 6.45 ppm (1H) and the multiplet at 6.64 ppm (2H). Based on literature, the C6 CH corresponds to the signal at 6.45 ppm, while the protons at C1 and C4 correspond to the signal at 6.64 ppm [35]. For the lazabemide moiety, two of the protons of the CH_2_–CH_2_ (C12/13) correspond to the multiplets at 3.20 and 3.75 ppm (the signals integrate for one proton each). The remaining two protons are most likely part of the multiplet at 3.30–3.36 ppm. The complex nature of these signals may be attributed to the chirality of the α-carbon, C8. The aromatic protons at C20 and C21 of the pyridyl ring correspond to the two doublets at 8.11 ppm (1H) and 8.03 ppm (1H). It is not clear why meta-coupling with C18 is not observed in this spectrum. The aromatic proton on C18 of the pyridyl ring corresponds to the signal at 8.69 ppm (1H). As mentioned above, in this spectrum, meta-coupling with C20 is not observed. In the ^1^H-NMR spectrum of the trifluoroacetic acid salt of the prodrug, meta-coupling was, however, observed between the signal at 8.11 ppm and the signal at 8.69 ppm (data not shown). The dihydrochloric acid of the prodrug contains eight exchangeable protons (NH, NH_3_^+^ and OH groups). These signals are most likely represented by the broad signals observed in the spectrum at 8.07 ppm (3H), 8.63 ppm (1H), 8.86 (1H) and 8.91 ppm (3H).

In the ^13^C-NMR spectrum, the carbonyl carbons (C10 and C15) are represented by the signals at 163.3 and 168.6 ppm. The methylene and methene carbons (C7, C8, C12 and C13) correspond to the signals at 36.6, 38.4, 38.6 and 54.1 ppm, respectively. Based on the DEPT 135° spectrum, the methylene CH_2_ groups at C7, C12 and C13 correspond to the signals at 36.6, 38.4 and 38.6 ppm, while the methene CH group (C8) is represented by the signal at 54.1 ppm. Aromatic CH carbons (6 carbons) are represented by signals at 115.7, 116.9, 120.2, 123.5, 137.6 and 147.1 ppm. Aromatic C carbons (5 carbons) are represented by signals at 125.8, 134.0, 144.5, 145.2 and 148.4 ppm.

Mass spectrometry reveals an experimental mass (*m*/*z*) 378.1089 Da, which best corresponds to an empirical formula of C_17_H_19_O_4_N_4_Cl (378.1095 Da). The difference (1.59 ppm) between the calculated and experimentally determined masses are indicative that the structure of the analysed compound corresponds to that of the l-dopa–lazabemide prodrug. Also of interest is the satellite signal at 380.10771, which corresponds to the l-dopa–lazabemide prodrug containing the ^37^Cl isotope.

### 2.2. Physicochemical Properties

#### 2.2.1. LogD

As shown in Table 1, the l-dopa–lazabemide prodrug may be viewed as hydrophilic at pH 6.4 since it displays logD < 0. As a general guide, logD values ranging from 0–3 are optimal for passive diffusion permeability and such compounds are expected to display good oral bioavailability and blood–brain barrier permeation [32]. At pH 7.4 and 7.8, the logD values are 0.199 and 0.319, respectively. This suggests that the l-dopa–lazabemide prodrug may permeate biological membranes by passive diffusion at these pH values. It is interesting to note that lazabemide also is a hydrophilic compound at the pH values evaluated. In fact, at pH 7.4 and 7.8, lazabemide is more hydrophilic than the l-dopa–lazabemide prodrug. In spite of this, lazabemide is an orally active MAO-B inhibitor which acts in the CNS. The pH values selected for this study fall in the range of 4.4–8 of the small intestine, the site of drug absorption [32].

#### 2.2.2. Solubility

The l-dopa–lazabemide prodrug was dissolved to a theoretical concentration of 6 mg/mL in water and buffer, and the concentration of material that dissolved was subsequently measured. These concentrations of the l-dopa–lazabemide prodrug in the water and buffer phases are shown in Table 2. The results show that the l-dopa–lazabemide prodrug is highly soluble in water and buffer since the concentrations recorded in water and buffer are, within experimental error, equal to 6 mg/mL. It may thus be concluded that the minimum solubility of the prodrug is 6 mg/mL, which may be viewed as good aqueous solubility.

#### 2.2.3. Ionisation Constant

The p*K*_a_ for the amine group of the l-dopa–lazabemide prodrug was found to be 7.25 ± 0.16. At physiological pH 7.4, approximately 59% of the prodrug will exist as the neutral uncharged species. Considering that the neutral species should display better membrane permeability than the ionised aminium, a relatively large fraction of the prodrug is available for passive diffusion. This result suggests that ionisation of the prodrug at pH 7.4 should not be a significant barrier for permeation through the blood–brain barrier. At the pH values found in the gastrointestinal tract (for example pH 5.5), however, only 1–2% of the prodrug will exist as the neutral uncharged species. This result suggests that ionisation of the prodrug in the gastrointestinal tract may represent a barrier for absorption into the systemic circulation. The pH 5.5 selected here to illustrate the degree of ionisation is representative of the pH in the duodenum and jejunum (4.4–6.6), where ionisation may be a significant barrier to drug absorption [32].

#### 2.2.4. Cell Viability

The toxicity of the l-dopa–lazabemide prodrug towards cultured cells was examined and compared to that of l-dopa. Although the prodrug consists of two known drugs, it may possess toxicity issues that are distinct from the two drugs of which it is composed. For this purpose, the MTT cell viability assay was used [36]. The MTT assay is a standard cell viability assay, measuring mitochondrial activity in live (metabolically viable) cells. Metabolically active mitochondria in viable cells have the ability to reduce MTT to coloured formazan crystals that can be quantitated spectrophotometrically. The presence of a toxic agent will reduce cell viability and thus limit the reduction of MTT to the formazan product. For this study, HeLa cells were selected. This selection was based on the high growth rate of HeLa cells and on the observation that these cells are frequently used to measure cell viability in the presence of toxic agents [37].

The percentages of viable cells after treatment with the l-dopa–lazabemide prodrug and l-dopa are shown in Table 3. It is evident from the results that at a concentration of 1 µM, neither the l-dopa–lazabemide prodrug nor l-dopa are toxic for the cultured cells. At a concentration of 10 µM, the l-dopa–lazabemide prodrug displays toxicity for the cultured cells (viability of 52.1%) while l-dopa is non-toxic. At concentrations of 100 µM, both the l-dopa–lazabemide prodrug and l-dopa display toxicity for the cultured cells with viabilities of 35% and 79%, respectively. These results indicate that the l-dopa–lazabemide prodrug is significantly more toxic for cultured cells at 10 µM and 100 µM than l-dopa. A possible explanation for the higher toxicity observed for the l-dopa–lazabemide prodrug is that the prodrug may permeate the cell membrane of the cultured cell more readily than l-dopa, and thus reach higher concentrations within the cytosol. This greater degree of intracellular exposure may lead to higher toxicity. More investigation to clarify this observation is, however, required.

#### 2.2.5. Passive Diffusion Permeability

The permeabilities of the l-dopa–lazabemide prodrug, lazabemide and l-dopa were evaluated via the parallel artificial membrane permeability assay (PAMPA) and are given in Table 4. From the data it is evident that l-dopa displays poor permeability at all pH values with logP_e_ values smaller than −7.99. Higher logP_e_ values are indicative of increased permeability. For example, propranolol, a drug considered to have good permeability, is reported to have a logP_e_ value of −4.4 at pH 6.8 [38]. In contrast to l-dopa, lazabemide displays better permeability with logP_e_ values of up to −5.67 at pH 7.8. It is interesting to note that the permeability of lazabemide increases with increasing pH. This is consistent with a lower degree of ionisation of this compound as pH increases. The l-dopa–lazabemide prodrug also displayed better permeability than l-dopa with the best permeability at pH 7.8 (logP_e_ = −7.33). As with lazabemide, the permeability of the prodrug increases with increasing pH, which is consistent with a lower degree of ionisation at higher pH values. Compared to propranolol and lazabemide, the l-dopa–lazabemide prodrug, however, displays poor permeability. The pH values selected here fall in the range of 4.4–8 of the small intestine, the site of drug absorption [32].

#### 2.2.6. Chemical Stability

The chemical stability of the l-dopa–lazabemide prodrug was determined in aqueous buffer. These measurements were done in order to determine if the prodrug may undergo nonenzymatic hydrolysis. Hydrolysis of the l-dopa–lazabemide prodrug will yield l-dopa and lazabemide. Such an event may be viewed as undesirable since premature hydrolysis prior to absorption may reduce the amount of the prodrug available for absorption into the systemic circulation. The prodrug should therefore be stable in aqueous solution. In general, nonenzymatic hydrolysis of the prodrug is undesirable, while enzymatic hydrolysis is the preferred method of activation.

The results of the chemical hydrolysis study show that the l-dopa–lazabemide prodrug is stable at pH 3.7–7.4, with no significant decrease of the peak area and concentration of the prodrug (up to 11 h) after the preparation of the solution. This indicates that the prodrug does not significantly hydrolyse at these pH values, and does not undergo other chemical transformations such as oxidation at these pH values. At a pH of 7.8, the concentration of the l-dopa–lazabemide prodrug, however, does decrease in a time-dependent manner. At 11 h after the preparation of the solution, the prodrug concentration has decreased to 90.3%. The decrease of the prodrug concentration was not accompanied by the appearance of the peak of lazabemide, which suggests that at higher pH values, the prodrug undergoes different chemical reactions than hydrolysis of the amide functionality.

#### 2.2.7. Plasma and Tissue Stability

Although the l-dopa–lazabemide prodrug should be stable in aqueous solution, the prodrug should undergo hydrolysis in plasma or tissue. It is preferable that the prodrug does not hydrolyse very quickly (within minutes) in plasma, but be relatively stable in order to provide enough exposure time of the prodrug to the blood–brain barrier. As mentioned above, if the prodrug is activated too quickly, a relatively small amount will gain access to the CNS. Slow enzymatic hydrolysis is thus the preferred method of activation of the l-dopa–lazabemide prodrug.

**Figure 5:**
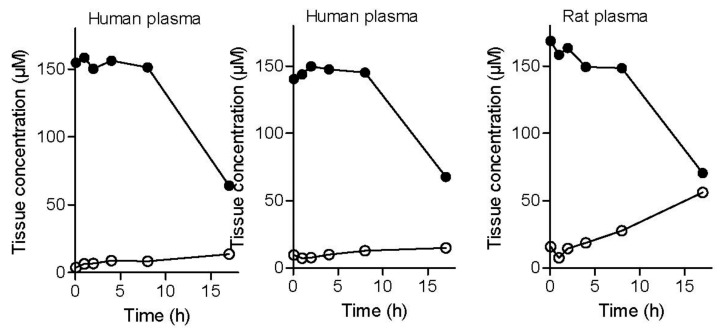
The metabolic stability of the l-dopa–lazabemide prodrug in human and rat plasma. Key: lazabemide (open circles); prodrug (filled circles). Values are given as mean (µM) ± standard deviation of triplicate determinations.

To evaluate plasma stability, three experiments were conducted. Two plasma stability experiments were carried out with human plasma (from different donors) and one study was carried out with rat plasma. The results of the plasma stability study shows that the l-dopa–lazabemide prodrug is stable in human plasma, with no significant decrease of the peak area and concentration of the prodrug at up to 8 h incubation in plasma (Figure 5). The appearance and increase of the peak of lazabemide, however, shows that small amounts of the prodrug are indeed hydrolysed. The increase of the concentration of lazabemide is time-dependent. At 17 h after the preparation of the spiked plasma samples, the prodrug concentration has decreased to 64–68 µM in human plasma and the concentration of lazabemide has increased to 13–15 µM. The relatively small concentration of lazabemide at 17 h indicates that lazabemide may undergo metabolism in the plasma, or that the prodrug also undergoes different chemical reactions other than hydrolysis of the amide functionality, thus not yielding lazabemide. Further investigation is necessary to clarify this point.

Similar results were obtained with rat plasma. The results document that the l-dopa–lazabemide prodrug is relatively stable in rat plasma, with a slow decrease of the concentration of the prodrug up to 8 h of incubation. After 17 h of incubation of the prodrug in rat plasma, the prodrug concentration has decreased to 70.5 µM. The degree of reduction in concentration of the prodrug is similar to that obtained with the human plasma. As with the human plasma, the decrease of prodrug concentration is accompanied with the appearance of the peak of lazabemide. Although more lazabemide is detected in rat plasma (56 µM) after 17 h than in human plasma, the concentration of lazabemide is still smaller than what would be expected if the prodrug has undergone only hydrolysis. The potential metabolism of lazabemide and/or the prodrug in rat plasma may explain this discrepancy. In drug design it has been found that drugs are typically less stable in the plasma of rodents compared to human plasma. Plasma stability can vary greatly among species, which makes extrapolation to the human difficult [32].

**Figure 6:**
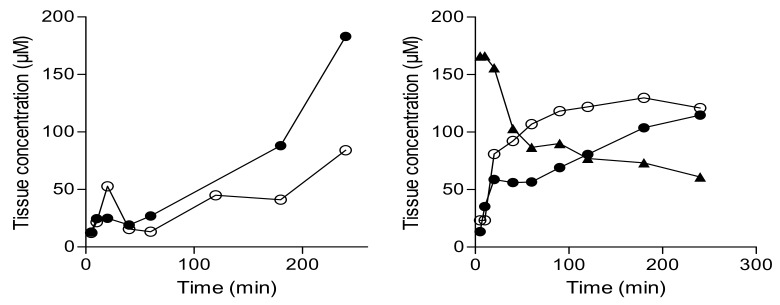
The metabolic stability of the l-dopa–lazabemide prodrug in rat brain (**left**) and liver (**right**) homogenates. Key: lazabemide (open circles); l-dopa (filled circles); prodrug (triangles). Values are given as mean (µM) ± standard deviation of triplicate determinations.

To evaluate tissue stability, experiments with both rat brain and liver homogenates were carried out. As in plasma, the concentration of the prodrug declines in a time-dependent manner in liver tissue with 37% remaining after 4 h incubation and an approximate *t*_½_ of 60 min (Figure 6). During this time, the concentrations of lazabemide and l-dopa increase in a time-dependent manner. In brain tissue homogenate, an increase in the concentrations of lazabemide and l-dopa was also observed with increased incubation time of the prodrug with the tissue. After 4 h incubation, the concentrations of lazabemide and l-dopa have increased 5-fold and 8-fold, respectively, compared to the concentrations at 5 min. These data indicate that, in both liver and brain tissue, the prodrug may indeed undergo activation to yield lazabemide and l-dopa.

### 2.3. In-Vivo Effect of the Prodrug on Brain Monoamines

**Figure 7:**
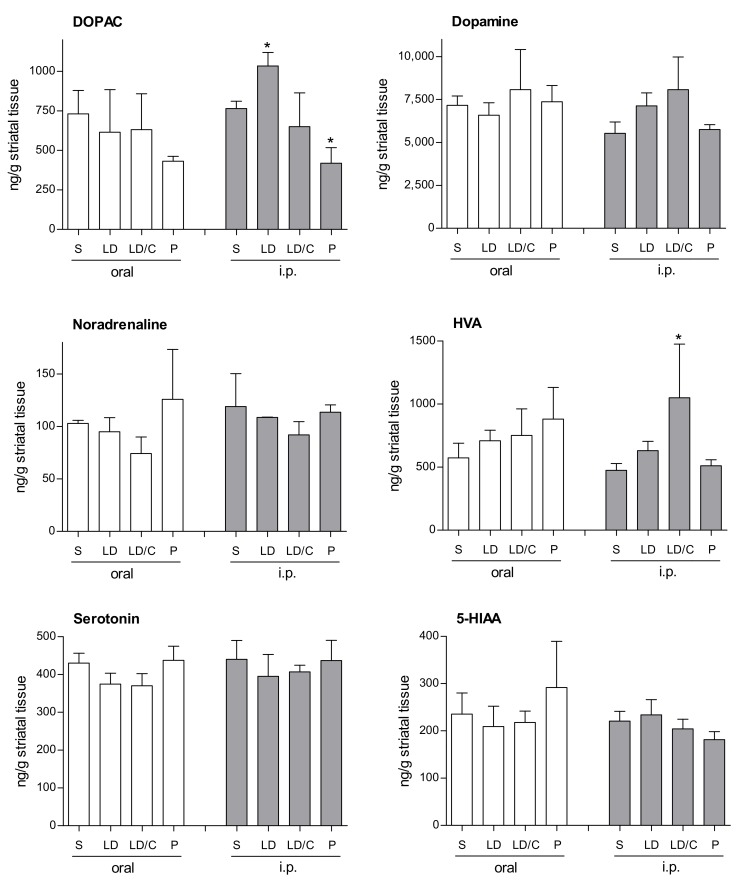
The concentrations of selected monoamines and metabolites in the striatum of mice following oral and intraperitoneal (ip) treatment with saline (S), l-dopa (LD), l-dopa and carbidopa (LD/C), or the l-dopa–lazabemide prodrug (P). Values are given as mean ± standard deviation with statistical comparisons to the saline-treated animals indicated (* *p* < 0.05; *n* = 3/group).

In preliminary experiments, the concentrations that the prodrug, lazabemide and l-dopa reach in the brain after oral and intraperitoneal administration were too low to be reliably detected by the HPLC/UV method developed in this study. The delivery of dopamine by the prodrug was thus indirectly assessed by measuring dopamine levels in the striatal tissue of mice following treatment with the prodrug. For this purpose, the measurement of dopamine metabolites and other monoamine neurotransmitters and their metabolites was also included. C57BL/6 mice were allocated into four groups (*n* = 3 mice/group) and each group was treated by oral gavage with either saline, l-dopa, l-dopa and carbidopa, or the l-dopa–lazabemide prodrug. l-Dopa and the l-dopa–lazabemide prodrug were given at a dose of 63.5 µmol/kg and carbidopa was given at 10 mg/kg, a dosage regimen similar to that reported in literature [39]. Another four groups (*n* = 3 mice/group) were treated in the same manner by intraperitoneal injection. The animals were sacrificed 60 min after treatment, the striata were dissected and the concentrations of dopamine, 3,4-dihydroxyphenylacetic acid (DOPAC), homovanillic acid (HVA), noradrenaline, serotonin and 5-hydroxyindoleacetic acid (HIAA) were measured [40]. These monoamines were selected since their levels (and those of their metabolites) may be affected by l-dopa and/or MAO inhibition. The in-vivo modulation of the striatal levels of these monoamines (and their metabolites) by the prodrug may give an indication of the potential of the prodrug as a therapeutic agent in Parkinson’s disease. The time point of 60 min was selected based on literature reports that after oral administration of l-dopa prodrugs to rats, the plasma half-life of l-dopa is 51–97 min [39]. In mice, l-dopa levels reached peak plasma levels 30–60 min after oral administration of l-dopa and carbidopa [41]. The results given in Figure 7 show that, while treatment with the prodrug does not enhance striatal dopamine levels, a significant reduction (compared to saline) in DOPAC is observed following intraperitoneal treatment. In this respect, one-way ANOVA (F(3,7) = 16.10; *p* = 0.0016) of the DOPAC concentration data revealed a significant effect of treatment, and post-hoc (Dunnett’s test) analysis indicated a significant decrease (*p* = 0.016) in DOPAC levels after intraperitoneal treatment with the prodrug versus saline-treated controls. Interestingly, intraperitoneal l-dopa treatment significantly enhanced (*p* = 0.049) DOPAC levels. None of the other monoamines measured were significantly altered by treatment. As will be discussed below, the observation that ip treatment with the prodrug significantly reduces DOPAC levels compared to saline, l-dopa and carbidopa/l-dopa treatment suggests that the prodrug may reduce dopamine metabolism in the striatum, most likely because of MAO-B inhibition by lazabemide. Also, the finding that ip treatment with l-dopa enhances DOPAC levels in the striatum suggests that l-dopa treatment supplements dopamine levels, which in turn is metabolised to yield increased concentrations of DOPAC. This finding is not observed with the prodrug, as lazabemide is expected to block the metabolic route (e.g., MAO-B) of dopamine conversion to DOPAC. As expected, serotonin and 5-HIAA levels are not altered by the prodrug since lazabemide is a MAO-B-specific inhibitor while serotonin is a specific substrate of MAO-A. The unresponsiveness of dopamine levels to treatment with the prodrug may be due to the fact that dopamine is a substrate of both MAO-A and MAO-B, and that the steady-state levels of dopamine may only be altered by inhibition of both isoforms. In this respect, when one isoform is inhibited in the brain, the other may take over its function [26].

## 3. Discussion

In the present study, an l-dopa–lazabemide prodrug is proposed to enhance the delivery of l-dopa to the brain after oral administration. As shown in the introduction, the prodrug is designed to possess enhanced permeability and metabolic stability compared to l-dopa. In addition, lazabemide, liberated by the activation of the prodrug, may protect against MAO-B-catalysed depletion of central dopamine, and possibly also reduce potentially neurotoxic species produced by the MAO catalytic cycle. An analysis of the properties of the prodrug shows that the prodrug possesses appropriate lipophilicity (logD) and solubility profiles for oral absorption, although passive diffusion permeability, as evaluated by PAMPA, is comparatively low. In spite of this, the prodrug displays higher toxicity to cultured cells than l-dopa, possibly indicating higher intracellular exposure of the prodrug as a result of better permeability. Further experiments show that the prodrug is stable towards hydrolysis in aqueous buffer and undergoes slow activation in plasma and tissue (liver and brain). Although oral and ip treatment of mice with the prodrug did not result in enhanced striatal dopamine levels, DOPAC levels were significantly depressed compared to saline, l-dopa and carbidopa/l-dopa treatment. This suggests that the prodrug may reduce dopamine metabolism, most likely as a result of MAO-B inhibition by lazabemide. After ip treatment with l-dopa, DOPAC levels are significantly increased, likely due to increased central dopamine in response to l-dopa. This behaviour is not observed with the prodrug, as lazabemide is expected to block the metabolic route leading from dopamine to DOPAC. Based on these data, further preclinical evaluation of the l-dopa–lazabemide prodrug should be undertaken with the aim of discovering prodrugs that may be advanced to the clinical stages of development.

## 4. Materials and Methods 

### 4.1. The Synthesis of Lazabemide

Lazabemide, as the free base, was synthesised from 5-chloro-2-cyanopyridine according to the published method [34].

### 4.2. The Synthesis of the l-Dopa–Lazabemide Prodrug

#### 4.2.1. Chemicals and Instrumentation

High-resolution mass spectra (HRMS) were recorded with a DFS high-resolution magnetic sector mass spectrometer (Thermo Electron Corporation, Waltham, MA, USA) in electron ionisation (EI) mode. Proton (^1^H)- and carbon (^13^C)-NMR spectra were recorded on a Bruker Avance III 600 spectrometer (Merck, Darmstadt, Germany) at a frequency of 600 MHz for ^1^H-NMR spectra and 150 MHz for ^13^C-NMR spectra. All chemical shifts are reported in parts per million (δ) and were referenced to the residual solvent signal (DMSO-*d*_6_: 2.50 and 39.52 ppm for ^1^H and ^13^C, respectively). Spin multiplicities are given as s (singlet), d (doublet), m (multiplet) or broad singlet (brs). All chemicals and reagents were obtained from Sigma-Aldrich (St. Louis, MO, USA) and were used without further purification.

#### 4.2.2. The Synthesis of l-Dopa(TBDMS)_2_

l-Dopa (8 mmol) was added to a solution of tert-butyldimethylsilyl chloride (TBDMS-Cl, 23.2 mmol) in acetonitrile (18 mL). The suspension was stirred and cooled to 0 °C, and 3.6 mL of 1,8-diazabicyclo[5.4.0]undec-7-ene (DBU, 24 mmol) was added. The reaction mixture was then stirred for 18 h at room temperature and was subsequently filtered. The precipitate was collected and dried at 50 °C to yield l-dopa(TBDMS)_2_ as a white powder in a yield of 37% [35].

#### 4.2.3. The Synthesis of Boc-l-Dopa(TBDMS)_2_

Di-*tert*-butyl dicarbonate (Boc_2_O, 8.8 mmol) was dissolved in 20 mL THF and added to a suspension l-dopa(TBDMS)_2_ (7.4 mmol) in 20 mL of water containing 8 mmol of NaHCO_3_. The reaction mixture was stirred for 24 h at room temperature and the THF was subsequently evaporated under reduced pressure. Water (10 mL) was added to the residue and the solution was extracted with diethyl ether (30 mL). The aqueous layer was acidified with citric acid (20%) to pH 5–6 and extracted three times with ethyl acetate (30 mL). Drying of the combined extracts over MgSO_4_ and removal of the solvent under reduced pressure yielded Boc-l-dopa(TBDMS)_2_ in a yield of 67% as an amorphous solid [35].

#### 4.2.4. The Conjugation of Protected l-Dopa with Lazabemide

Protected l-dopa [Boc-l-dopa(TBDMS)_2_] (2 mmol), lazabemide (2 mmol) and 2 mmol of the BOP reagent [(1*H*-benzotriazol-1-yloxy)tris(dimethylamino)phosphonium hexafluorophosphate] were added to 15 mL dichloromethane. *N*-Methylmorpholine (NMM, 4 mmol) was added to the reaction mixture, and the reaction was stirred for 24 h at room temperature. The dichloromethane was evaporated under reduced pressure and 5 mL of water added to the residue. The residue was subsequently extracted with ethyl acetate (35 mL). The organic phase was washed successively with 2% citric acid, water, a 3% aqueous NaHCO_3_ solution and water, and was finally dried over MgSO_4_. After concentration of the solution under reduced pressure the crude substance was incubated overnight upon which it solidified. The product, the protected l-dopa–lazabemide prodrug (Boc-l-dopa(TBDMS)_2_-lazabemide) was obtained in a yield of 80% [35].

#### 4.2.5. Removal of the TBDMS and Boc Protective Groups from Boc-l-Dopa(TBDMS)_2_-lazabemide

The protected l-dopa–lazabemide prodrug (Boc-l-dopa(TBDMS)_2_-lazabemide) (150 mg) was dissolved in 1.5 mL HCl solution (4 M in dioxane) and stirred for 48 h at 55 °C. The deprotecting agent was evaporated under reduced pressure and the residue was washed three times with diethyl ether (30 mL). In each instance, the diethyl ether was removed under reduced pressure. A white crystalline product, as the hydrochloric acid salt, was obtained in good yield (61 mg; 32.2%) [35]. ^1^H-NMR (Bruker Avance III 600, DMSO-*d*_6_) δ 2.75–2.78 (m, 1H), 2.86–2.89 (m, 1H), 3.20 (m, 1H), 3.30–3.36 (m, 3H), 3.75 (m, 1H), 6.45 (d, 1H, *J* = 7.9 Hz), 6.64 (m, 2H), 8.03 (d, 1H, *J* = 8.3 Hz), 8.07 (brs, 3H), 8.11 (d, 1H, *J* = 8.3 Hz), 8.63 (s, 1H), 8.69 (s, 1H), 8.86 (m, 1H), 8.91 (brs, 3H). ^13^C-NMR (Bruker Avance III 600, DMSO-*d*_6_) δ 36.6, 38.4, 38.6, 54.1, 115.7, 116.9, 120.2, 123.5, 125.8, 134.0, 137.6, 144.5, 145.2, 147.1, 148.4, 163.3, 168.6. EI–HRMS *m*/*z* calcd. for C_17_H_19_O_4_N_4_Cl (M^+^) 378.1095, found 378.1089.

### 4.3. Determination of Physicochemical and Biological Properties

#### 4.3.1. Materials and Instrumentation

UV-Vis spectrophotometry was carried out with a Shimadzu MultiSpec-1501 UV-Vis photodiode array spectrophotometer (Kyoto, Japan). A Multiscan RC UV/Vis plate reader (Labsystems, CO, USA) was used to measure the absorbances in 96-well microplates. For potentiometric titration, a Hanna HI1230B general-purpose electrode was employed and standardised KOH (0.1 N) was obtained from Merck (Darmstadt, Germany). Precoated parallel artificial membrane permeability assay (PAMPA) plate systems were obtained from BD Biosciences (Franklin Lakes, NJ, USA) while Phree phospholipid-removal tubes were from Phenomonex (Torrance, CA, USA). MTT (3-(4,5-dimethylthiazol-2-yl)-2,5-diphenyltetrazolium bromide) and phosphate-buffered saline (PBS) were obtained from Sigma-Aldrich. Cell culture media (Dulbecco’s Modified Eagle Medium; DMEM), fetal bovine serum, penicillin (10,000 units/mL)/streptomycin (10 mg/mL), fungizone (250 µg/mL) and trypsin/EDTA (0.25%/0.02%) were from Gibco (Johannesburg, South Africa). 24-Well and 96-well plates were from Corning (Port Elizabeth, South Africa) while sterile syringe filters (0.22 µm) were obtained from Pall Corporation Life Sciences (Midrand, South Africa).

#### 4.3.2. Ethics Consideration

The treatment of the animals was conducted at the Vivarium at the Potchefstroom campus of the North-West University (NWU). Animals were bred, supplied and housed at the Vivarium (SAVC reg No. FR15/13458; SANAS GLP compliance No. G0019) of the Preclinical Drug Development Platform of the NWU. Experiments were approved by the AnimCare animal research ethics committee (NHREC reg. number AREC-130913-015) at the NWU. All animals were maintained and procedures performed in accordance with the code of ethics in research, training and testing of drugs in South Africa, and complied with national legislation. Ethical approval for the collection and use of human blood was obtained from the Research Ethics Committee, NWU. Ethics approval numbers: NWU-00325-15-A5, NWU-00326-15-A5, NWU-00056-11-S5.

#### 4.3.3. Shake-Flask Method for logD Determination

*n*-Octanol (analytical reagent from Sigma-Aldrich) and the appropriate buffer were mutually saturated in a separatory funnel. Potassium phosphate buffer (100 mM) at pH 6.4, 7.4 and 7.8 served as the aqueous buffer phases. In a 10 mL glass vessel, 4 mL of *n*-octanol was placed followed by 4 mL of buffer containing 2 mM of the l-dopa–lazabemide prodrug. The vessels were shaken by hand for 5 min and centrifuged at 4000× *g* for 10 min. The concentrations of the l-dopa–lazabemide prodrug in the *n*-octanol and buffer phases were determined by employing the molar extinction coefficients (at 276 nm) recorded in *n*-octanol and in each of the three buffers (pH 6.4, 7.4 and 7.8) employed. The molar extinction coefficient of the prodrug in *n*-octanol was found to be 12,493 M^−1^. The molar extinction coefficients of the prodrug in the three buffers were similar and were found to be 13,583 M^−1^. The logD value at a given pH is equal to the logarithm of the octanol/buffer partition coefficients (D). The partition coefficient is the ratio of the concentration of the l-dopa–lazabemide prodrug in the *n*-octanol phase to the buffer phase. LogD values are given as mean ± standard deviation of triplicate determinations. Using the same protocol as above, the logD values at pH 6.4, 7.4 and 7.8 for lazabemide were also determined. The molar extinction coefficient of lazabemide in *n*-octanol was found to be 9666 M^−1^. The molar extinction coefficients of lazabemide in the three buffers were similar and was found to be 11,159 M^−1^.

#### 4.3.4. Determination of Solubility

To determine the solubility of the l-dopa–lazabemide prodrug, 18 mg of the drug was placed into a polypropylene tube and 3 mL water or potassium phosphate buffer (pH 7.4) was added. This yielded a theoretical concentration of 6.0 mg/mL. The tubes were agitated for 24 h in a water bath at 37 °C and were subsequently centrifuged at 16,000× *g* for 10 min. The samples were filtered through a 0.22 µm syringe filter and diluted 150-fold into water or potassium phosphate buffer (100 mM, pH 7.4). The absorbances of the resulting solutions were recorded at a wavelength of maximal absorbance of 276 nm. The concentration of the l-dopa–lazabemide prodrug was determined by employing the molar extinction coefficient measured in water (13,956 M^−1^) and that cited above (13,583 M^−1^) for potassium phosphate buffer (100 mM, pH 7.4). The solubility values are given as mean ± standard deviation of triplicate determinations.

#### 4.3.5. Determination of Ionisation Constant, p*K*_a_

A 10 mM solution of the l-dopa–lazabemide prodrug in 10 mL water was prepared and potentiometrically titrated with a standardised solution of KOH (0.1 N). The prodrug was titrated in 0.1 mL increments and the pH was recorded after each addition. A total of 1 mL KOH was added to fully neutralise the primary amine group of the prodrug. The results were subsequently tabulated according to the method described in literature, and the p*K*_a_ value was calculated [42].

#### 4.3.6. Determination of Toxicity towards Cultured Cells

HeLa cells were maintained in 250 cm^2^ flasks in 30 mL Dulbecco’s Modified Eagle Medium (DMEM) containing 10% fetal bovine serum, 1% penicillin (10,000 units/mL)/streptomycin (10 mg/mL) and 0.1% fungizone (250 µg/mL). The cells were incubated at 37 °C in an atmosphere of 10% CO_2_. Once confluent, the cells were seeded at 500,000 cells/well in 24-well plates and incubated for 24 h. A volume of 3 mL trypsin/EDTA (0.25%/0.02%) was used to facilitate cell detachment and the counting of cells was done with a haemocytometer. The wells were subsequently rinsed with 0.5 mL DMEM containing no fetal bovine serum. A volume of 0.99 mL DMEM (containing no fetal bovine serum) was subsequently added to each well followed by 10 µL of the test drug. Stock solutions of the test drugs were prepared in deionised water and sterilised via a 0.22 µm syringe filter. In each 24-well plate, wells were reserved as positive controls (100% cell death via lyses with 0.3% formic acid) and negative controls (0% cell death as a result of no treatment). The well-plates were incubated for a further 24 h after which the culture medium was aspirated from the cells. The wells were washed twice with 0.5 mL/well phosphate-buffered saline (PBS) and 200 µL of 0.05% MTT reagent (3-(4,5-dimethylthiazol-2-yl)-2,5-diphenyltetrazolium bromide) in PBS was added to each well. The well-plates were incubated at 37 °C for 2 h in the dark, the MTT reagent was aspirated from the wells and 250 µL isopropanol was added to each well. The well-plates were then incubated at room temperature for 5 min to dissolve the purple formazan crystals, whereafter 100 µL of the isopropanol phase was transferred to a 96-well plate. The absorbance of each isopropanol phase was measured spectrophotometrically at 560 nm. The effect on cell viability of each drug (l-dopa and the l-dopa–lazabemide prodrug) was tested in triplicate [37].

#### 4.3.7. High-Performance Liquid Chromatography (HPLC)

To measure the concentrations of the l-dopa–lazabemide prodrug, lazabemide and l-dopa in buffer and biological matrices, an HPLC method was developed. The HPLC system consisted of an Agilent 1200 series HPLC system with a binary gradient pump, autosampler and vacuum degasser coupled to an Agilent 1200 series variable-wavelength detector (Santa Clara, CA, USA). The analyses were carried out with Venusil XBP C18 column (4.60 mm × 150 mm, 5 μm, Bonna-Agela Technologies, Wilmington, DE, USA). For the analyses of the l-dopa–lazabemide prodrug (retention time 5.2 min) and lazabemide (retention time 3.3 min), the mobile phase consisted of 60% sodium acetate buffer (50 mM, pH 4.7) and 40% methanol at a flow rate of 1 mL/min. The effluent was monitored at 276 nm. For the analysis of l-dopa (retention time 3.0 min), the mobile phase consisted of 95% sodium acetate buffer (50 mM, pH 4.7) and 5% methanol at a flow rate of 1 mL/min. The effluent was monitored at 280 nm. A volume of 20 µL of the samples was injected into the HPLC system.

#### 4.3.8. Determination of Passive Diffusion Permeability

The precoated parallel artificial membrane permeability assay (PAMPA) plate system was obtained from BD Biosciences. Prior to use, the pre-coated PAMPA plates were warmed to room temperature for at least 30 min. Once warmed to room temperature, the plate system was used within 24 h. A volume of 300 µL of the test compound solution in potassium phosphate buffer (100 mM, pH 6.4, 7.4 and 7.8) or in sodium acetate buffer (50 mM, pH 3.7, 4.7 and 5.7) was added per well in the receiver/donor plate (bottom). The final concentration of the test compounds was 200 µM in each well. A volume of 200 µL of potassium phosphate buffer (100 mM, pH 6.4, 7.4 and 7.8) or sodium acetate buffer (50 mM, pH 3.7, 4.7 and 5.7) was then added per well to the filter/acceptor plate (top). The filter plate was placed on the receiver plate by slowly lowering the precoated PAMPA plate until it was seated on the receiver plate. The assembly was incubated at room temperature for 5 h. The precoated PAMPA plate and the receiver plate were separated. The compound concentrations were determined using the HPLC system described above. The permeability values are given as mean ± standard deviation of triplicate determinations [38].

#### 4.3.9. Determination of Chemical Stability

To determine the chemical stability of the l-dopa–lazabemide prodrug, a 10 mM stock solution of the prodrug in aqueous buffer was prepared. Potassium phosphate buffer (100 mM, pH 6.4, 7.4 and 7.8) or sodium acetate buffer (50 mM, pH 3.7, 4.7 and 5.7) served as the aqueous buffer phases. The stock solution was immediately diluted to 50 µM and the resulting solution was analysed by the HPLC method described above. The first injection served as reference point, after which the same solution was again injected at 1 h, 3 h, 6 h, 9 h and 11 h. During this time, the test solution remained at room temperature in the autosampler tray. The stabilities of the prodrug in the selected buffer phases are reported as the percentage mean ± standard deviation of the first injection, which served as reference point.

#### 4.3.10. Determination of Plasma and Tissue Stability

Plasma was collected in 0.5 mL MiniCollect^®^ heparin plasma tubes (Greiner Bio-One, Kremsmünster, Austria) and centrifuged at 5500 rpm. Liver and brain tissue were collected, placed on ice and rinsed with 0.25 M sucrose dissolved in water. Sucrose solution was added to the tissue at a ratio 1:3 (*w*/*v*), the tissues were cut into small pieces using scissors and homogenised by 2 passes (60 s each) using a Potter–Elvehjem homogeniser (1200 rev/min, Potter–Elvehjem, Kennesaw, GA, USA) [43]. Care was taken to cool the homogeniser in ice during this procedure. The plasma was stored at −80 °C until the day of the experiment while the tissue homogenate was used immediately. To determine the plasma and tissue stabilities of the l-dopa–lazabemide prodrug, a 10 mM stock solution of the prodrug in MilliQ water was prepared. The stock solution was immediately diluted to approximately 200 µM into plasma or tissue homogenate (preheated to 37 °C) and the resulting mixtures were analysed by the HPLC method described above. For this purpose, 100 µL of the spiked plasma or tissue homogenate was added to 300 µL acetonitrile (containing formic acid 1%) in a Phree tube. The tube was vortexed for 2 min and filtered under vacuum (9–10 mmHg). The collected solvent was evaporated under a stream of air, and the residue was redissolved in 100 µL methanol. A volume of 300 µL water was added and 20 µL of the resulting solution was analysed by the HPLC system. The first injection occurred at 5 min after preparation of the spiked plasma or tissue sample, after which aliquots of the same plasma sample or tissue homogenate were again subjected to the Phree work-up and injected at various time points. During this time, the plasma solution and tissue homogenate remained at 37 °C in a water bath. The stabilities of the prodrug are reported as the mean ± standard deviation of the remaining concentration of the prodrug in the plasma sample or tissue homogenate. The concentration of lazabemide was also determined.

### 4.4. Animal Studies

Male C57BL/6 mice were weighed and allocated into 4 groups of 3 mice each. The 4 groups were treated by oral gavage with saline, l-dopa, l-dopa and carbidopa, and the l-dopa–lazabemide prodrug. l-Dopa and the l-dopa–lazabemide prodrug were given at a dose of 63.5 µmol/kg and carbidopa was given at 10 mg/kg. A second set of 4 groups (*n* = 3) were treated in the same manner by intraperitoneal injection. All solutions were prepared in sterile saline and administered at volumes of 0.1 mL for oral gavage and 0.2 mL for intraperitoneal injection. The animals were euthanised 60 min after treatment and the striata were dissected and stored at −80 °C until the day of the experiment. The concentrations of dopamine, 3,4-dihydroxyphenylacetic acid (DOPAC), homovanillic acid (HVA), noradrenaline, serotonin and 5-hydroxyindoleacetic acid (5-HIAA) were determined according to the published protocol, and are reported as mean ± standard deviation of each group [40]. Prism version 5 (GraphPad Software, San Diego, CA, USA) was used for the statistical analyses and for the preparation of graphical presentations. Data were analysed by one-way analysis of variance (ANOVA) across all groups, and were subsequently subjected to the Dunnett’s post-hoc test to determine statistical differences between the means. A *p*-value of <0.05 indicates a statistically significant difference.

## Figures and Tables

**Table 1:** The logD values of the l-dopa–lazabemide prodrug and lazabemide at different pH values.

pH Value	LogD—Prodrug	LogD—Lazabemide
6.4	–4.58 ± 0.002	–1.18 ± 0.032
7.4	0.199 ± 0.002	–0.68 ± 0.019
7.8	0.319 ± 0.003	–0.63 ± 0.009

Values are given as mean ± standard deviation of triplicate determinations.

**Table 2:** The solubility of the l-dopa–lazabemide prodrug in water and aqueous buffer at pH 7.4.

Medium	Amount Dissolved (μg/mL)	Amount Measured (μg/mL)
Water	6000	6277 ± 747
pH 7.4	6000	6142 ± 730

Values are given as mean ± standard deviation of triplicate determinations.

**Table 3:** The percentage of viable cells remaining after treatment with the l-dopa–lazabemide prodrug and l-dopa.

	Concentration of the Test Drug		
	1 µM	10 µM	100 µM
l-Dopa	102 ± 4.19	105 ± 5.24	79.3 ± 13.0
l-Dopa–lazabemide prodrug	112 ± 12.0	52.1 ± 14.0	34.9 ± 7.94

Values are given as mean (percentage) ± standard deviation of triplicate determinations.

**Table 4:** The permeability (P_e_) of the l-dopa–lazabemide prodrug, lazabemide and l-dopa at selected pH values.

	Permeability (cm/s) Expressed as LogP_e_					
	pH 3.7	pH 4.7	pH 5.7	pH 6.4	pH 7.4	pH 7.8
l-Dopa	−7.99 ± 0.28	−8.05 ± 0.19	−8.22 ± 0.14	−8.62 ± 0.87	−8.17 ± 0.56	−8.56 ± 0.07
Prodrug	−8.02 ± 0.39	−7.81 ± 0.18	−7.71 ± 0.14	−7.71 ± 0.06	−7.53 ± 0.04	−7.33 ± 0.30
Lazabemide	−7.37 ± 0.24	−7.06 ± 0.05	−6.83 ± 0.18	−6.45 ± 0.28	−5.79 ± 0.03	−5.67 ± 0.06

Values are given as mean ± standard deviation of triplicate determinations.

## Supplementary Materials

The following are available online. Mass spectra, ^1^H-NMR, ^13^C-NMR and DEPT-135° spectra.

## References

[1] Dauer W., Przedborski S. (2003). Parkinson’s disease: Mechanisms and models. Neuron.

[2] Freitas M.E., Ruiz-Lopez M., Fox S.H. (2016). Novel levodopa formulations for Parkinson’s disease. CNS Drugs.

[3] Poewe W., Antonini A. (2015). Novel formulations and modes of delivery of levodopa. Mov. Disord..

[4] Di Stefano A., Sozio P., Cerasa L.S., Iannitelli A. (2011). l-Dopa prodrugs: An overview of trends for improving Parkinson’s disease treatment. Curr. Pharm. Des..

[5] Di Stefano A., Sozio P., Cerasa L.S. (2008). Antiparkinson prodrugs. Molecules.

[6] Contin M., Martinelli P. (2010). Pharmacokinetics of levodopa. J. Neurol..

[7] Seeberger L.C., Hauser R.A. (2015). Carbidopa levodopa enteral suspension. Expert Opin. Pharmacother..

[8] Nutt J.G., Fellman J.H. (1984). Pharmacokinetics of levodopa. Clin. Neuropharmacol..

[9] Tohgi H., Abe T., Kikuchi T., Takahashi S., Nozaki Y. (1991). The significance of 3-*O*-methyldopa concentrations in the cerebrospinal fluid in the pathogenesis of wearing-off phenomenon in Parkinson’s disease. Neurosci. Lett..

[10] Learmonth D.A., Palma P.N., Vieira-Coelho M.A., Soares-da-Silva P. (2004). Synthesis, biological evaluation, and molecular modeling studies of a novel, peripherally selective inhibitor of catechol-*O*-methyltransferase. J. Med. Chem..

[11] Nissinen E., Linden I.B., Schultz E., Pohto P. (1992). Biochemical and pharmacological properties of a peripherally acting catechol-*O*-methyltransferase inhibitor entacapone. Naunyn Schmiedebergs Arch. Pharmacol..

[12] Shoulson I., Glaubiger G.A., Chase T.N. (1975). On-off response. Clinical and biochemical correlations during oral and intravenous levodopa administration in parkinsonian patients. Neurology.

[13] Nutt J.G. (1987). On-off phenomenon: Relation to levodopa pharmacokinetics and pharmacodynamics. Ann. Neurol..

[14] Leenders K.L., Poewe W.H., Palmer A.J., Brenton D.P., Frackowiak R.S. (1986). Inhibition of l-[^18^f]fluorodopa uptake into human brain by amino acids demonstrated by positron emission tomography. Ann. Neurol..

[15] Nutt J.G., Woodward W.R., Hammerstad J.P., Carter J.H., Anderson J.L. (1984). The “on-off” phenomenon in Parkinson’s disease. Relation to levodopa absorption and transport. N. Engl. J. Med..

[16] Lewitt P.A., Ellenbogen A., Chen D., Lal R., McGuire K., Zomorodi K., Luo W., Huff F.J. (2012). Actively transported levodopa prodrug xp21279: A study in patients with Parkinson disease who experience motor fluctuations. Clin. Neuropharmacol..

[17] LeWitt P.A., Huff F.J., Hauser R.A., Chen D., Lissin D., Zomorodi K., Cundy K.C. (2014). Double-blind study of the actively transported levodopa prodrug xp21279 in Parkinson’s disease. Mov. Disord..

[18] Cesura A.M., Muggli-Maniglio D., Lang G., Imhof R., Da Prada M. (1990). Monoamine oxidase inhibition by moclobemide and 2-amino-ethyl carboxamide derivatives: Mode of action and kinetic characteristics. J. Neural Transm. Suppl..

[19] Cesura A.M., Borroni E., Gottowik J., Kuhn C., Malherbe P., Martin J., Richards J.G. (1999). Lazabemide for the treatment of Alzheimer’s disease: Rationale and therapeutic perspectives. Adv. Neurol..

[20] Binda C., Li M., Hubálek F., Restelli N., Edmondson D.E., Mattevi A. (2003). Insights into the mode of inhibition of human mitochondrial monoamine oxidase B from high-resolution crystal structures. Proc. Natl. Acad. Sci. USA.

[21] Edmondson D.E., Mattevi A., Binda C., Li M., Hubálek F. (2004). Structure and mechanism of monoamine oxidase. Curr. Med. Chem..

[22] Dingemanse J., Wood N., Jorga K., Kettler R. (1997). Pharmacokinetics and pharmacodynamics of single and multiple doses of the MAO-B inhibitor lazabemide in healthy subjects. Br. J. Clin. Pharmacol..

[23] Fowler J.S., Volkow N.D., Logan J., Schlyer D.J., MacGregor R.R., Wang G.J., Wolf A.P., Pappas N., Alexoff D., Shea C. (1993). Monoamine oxidase B (MAO B) inhibitor therapy in Parkinson’s disease: The degree and reversibility of human brain MAO B inhibition by Ro 19 6327. Neurology.

[24] Youdim M.B., Edmondson D., Tipton K.F. (2006). The therapeutic potential of monoamine oxidase inhibitors. Nat. Rev. Neurosci..

[25] Finberg J.P., Wang J., Bankiewicz K., Harvey-White J., Kopin I.J., Goldstein D.S. (1998). Increased striatal dopamine production from l-DOPA following selective inhibition of monoamine oxidase B by R(+)-*N*-propargyl-1-aminoindan (rasagiline) in the monkey. J. Neural Transm. Suppl..

[26] Youdim M.B., Bakhle Y.S. (2006). Monoamine oxidase: Isoforms and inhibitors in Parkinson’s disease and depressive illness. Br. J. Pharmacol..

[27] Fowler J.S., Volkow N.D., Wang G.J., Logan J., Pappas N., Shea C., MacGregor R. (1997). Age-related increases in brain monoamine oxidase B in living healthy human subjects. Neurobiol. Aging.

[28] Muller T. (2016). Emerging approaches in Parkinson’s disease—Adjunctive role of safinamide. Ther. Clin. Risk Manag..

[29] Robakis D., Fahn S. (2015). Defining the role of the monoamine oxidase-B inhibitors for Parkinson’s disease. CNS Drugs.

[30] Mason R.P., Olmstead E.G., Jacob R.F. (2000). Antioxidant activity of the monoamine oxidase B inhibitor lazabemide. Biochem. Pharmacol..

[31] Silverman R.B. (2004). The Organic Chemistry of Drug Design and Drug Action.

[32] Kerns E.H., Di L. (2008). Drug-Like Properties: Concepts, Structure Design and Methods: From ADME to Toxicity Optimization.

[33] Berlin I., Aubin H.J., Pedarriosse A.M., Rames A., Lancrenon S., Lagrue G. (2002). Lazabemide in Smoking Cessation Study Investigators. Lazabemide, a selective, reversible monoamine oxidase B inhibitor, as an aid to smoking cessation. Addiction.

[34] Soriato G., Focati M.P., Brescello R., Cotarca L., Giovanetti R. (2008). Pharmaceutical Preparations of Crystalline Lazabemide.

[35] Nakonieczna L., Przychodzeń W., Chimiak A. (1994). A new convenient route for the synthesis of dopa peptides. Liebigs Ann. Chem..

[36] Mosmann T. (1983). Rapid colorimetric assay for cellular growth and survival: Application to proliferation and cytotoxicity assays. J. Immunol. Methods.

[37] Brink C.B., Pretorius A., van Niekerk B.P., Oliver D.W., Venter D.P. (2008). Studies on cellular resilience and adaptation following acute and repetitive exposure to ozone in cultured human epithelial (HeLa) cells. Redox Rep..

[38] Wohnsland F., Faller B. (2001). High-throughput permeability pH profile and high-throughput alkane/water log P with artificial membranes. J. Med. Chem..

[39] Zhou T., Hider R.C., Jenner P., Campbell B., Hobbs C.J., Rose S., Jairaj M., Tayarani-Binazir K.A., Syme A. (2010). Design, synthesis and biological evaluation of l-dopa amide derivatives as potential prodrugs for the treatment of Parkinson’s disease. Eur. J. Med. Chem..

[40] Harvey B.H., Brand L., Jeeva Z., Stein D.J. (2006). Cortical/hippocampal monoamines, HPA-axis changes and aversive behavior following stress and restress in an animal model of post-traumatic stress disorder. Physiol. Behav..

[41] Kaenmaki M., Tammimaki A., Garcia-Horsman J.A., Myohanen T., Schendzielorz N., Karayiorgou M., Gogos J.A., Mannisto P.T. (2009). Importance of membrane-bound catechol-*O*-methyltransferase in l-dopa metabolism: A pharmacokinetic study in two types of COMT gene modified mice. Br. J. Pharmacol..

[42] Albert A., Serjeant E.P. (1984). The Determination of Ionization Constants. A Laboratory Manual.

[43] Barrow P.C., Holt S.J. (1971). Differences in distribution of esterase between cell fractions of rat liver homogenates prepared in various media. Relevance to the lysosomal location of the enzyme in the intact cell. Biochem. J..

