# Supplementary material for: The Design and Evaluation of an l-Dopa–Lazabemide Prodrug for the Treatment of Parkinson’s Disease
Source: Molecules. 2017 Nov 27;22(12):2076. doi: 10.3390/molecules22122076 (PMC6150007; doi:10.3390/molecules22122076)

Supplementary material

Figure S1. High resolution mass spectrum of the L-dopa-lazabemide prodrug.

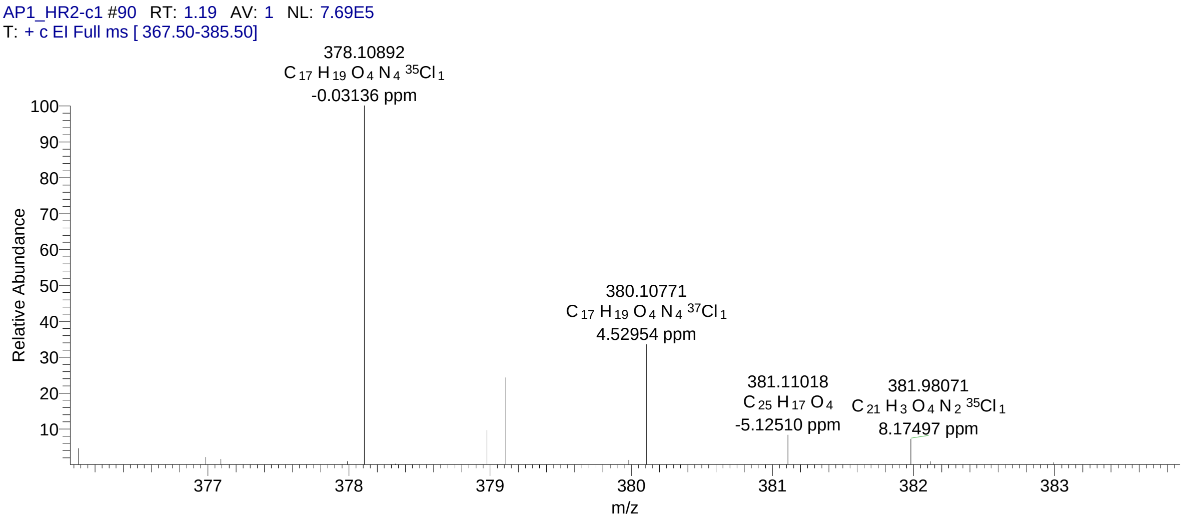

Figure S2. NMR spectra of the L-dopa-lazabemide prodrug

^{1}H NMR

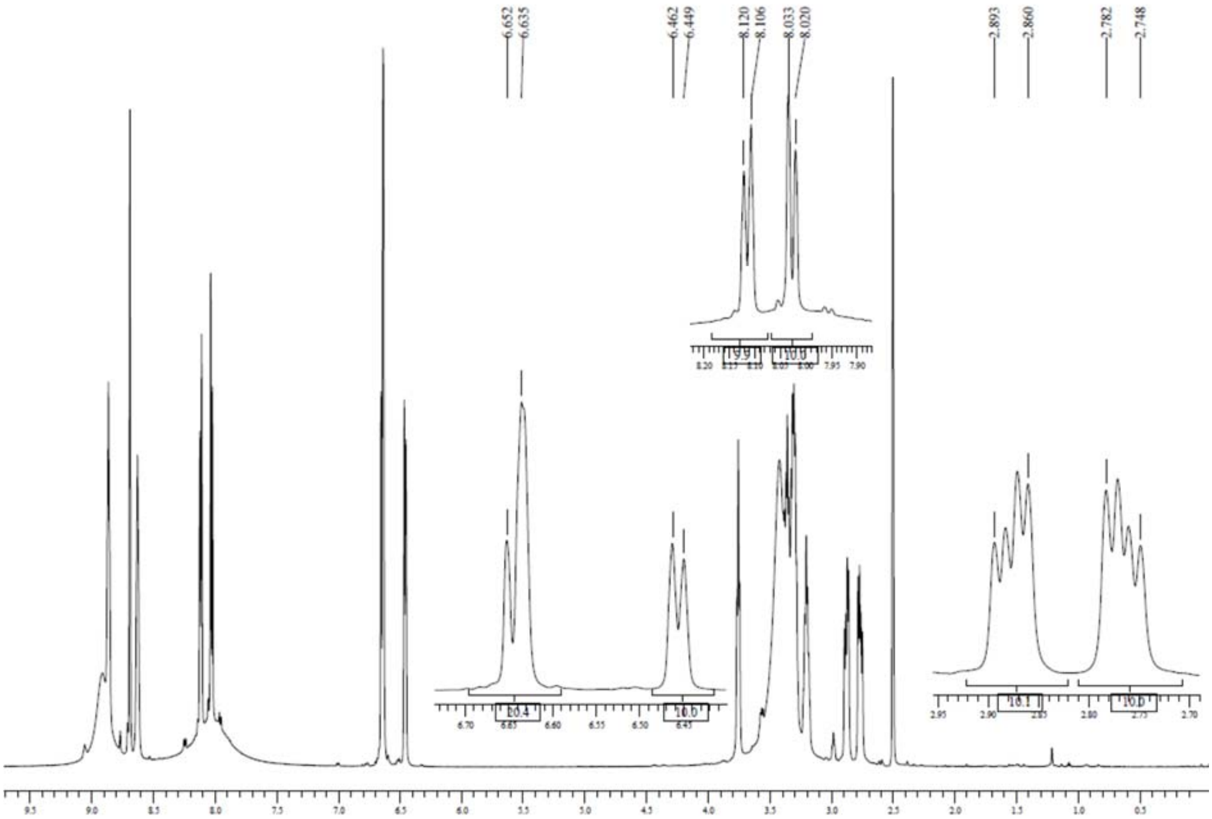

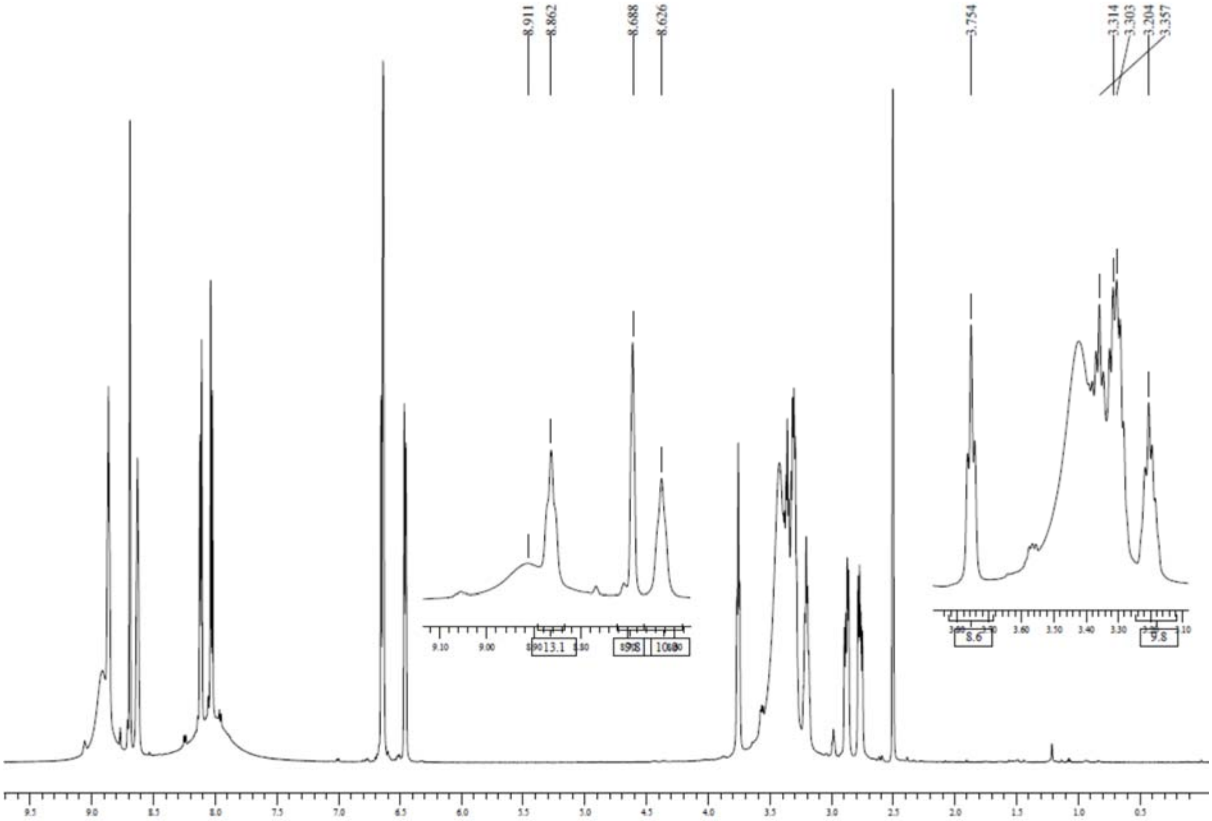

^{13}C NMR

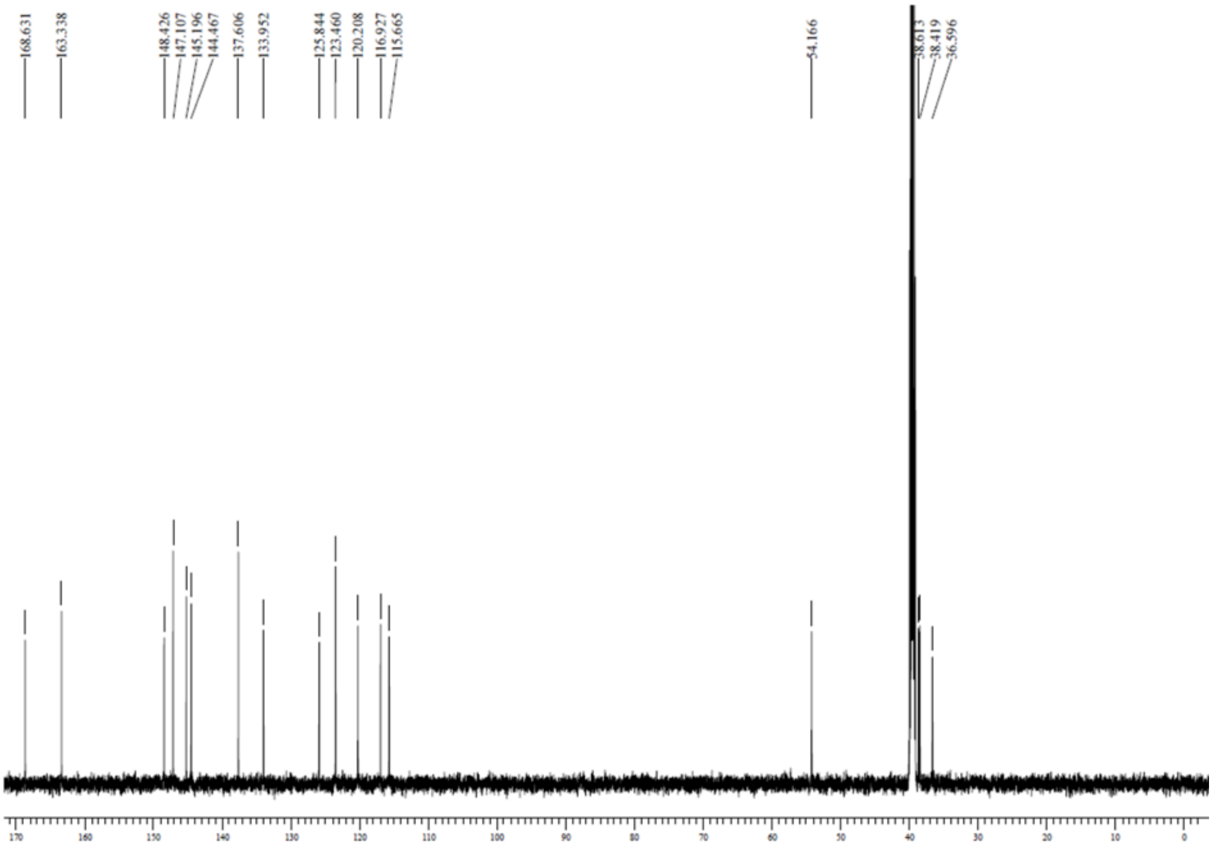

^{13}C DEPT135

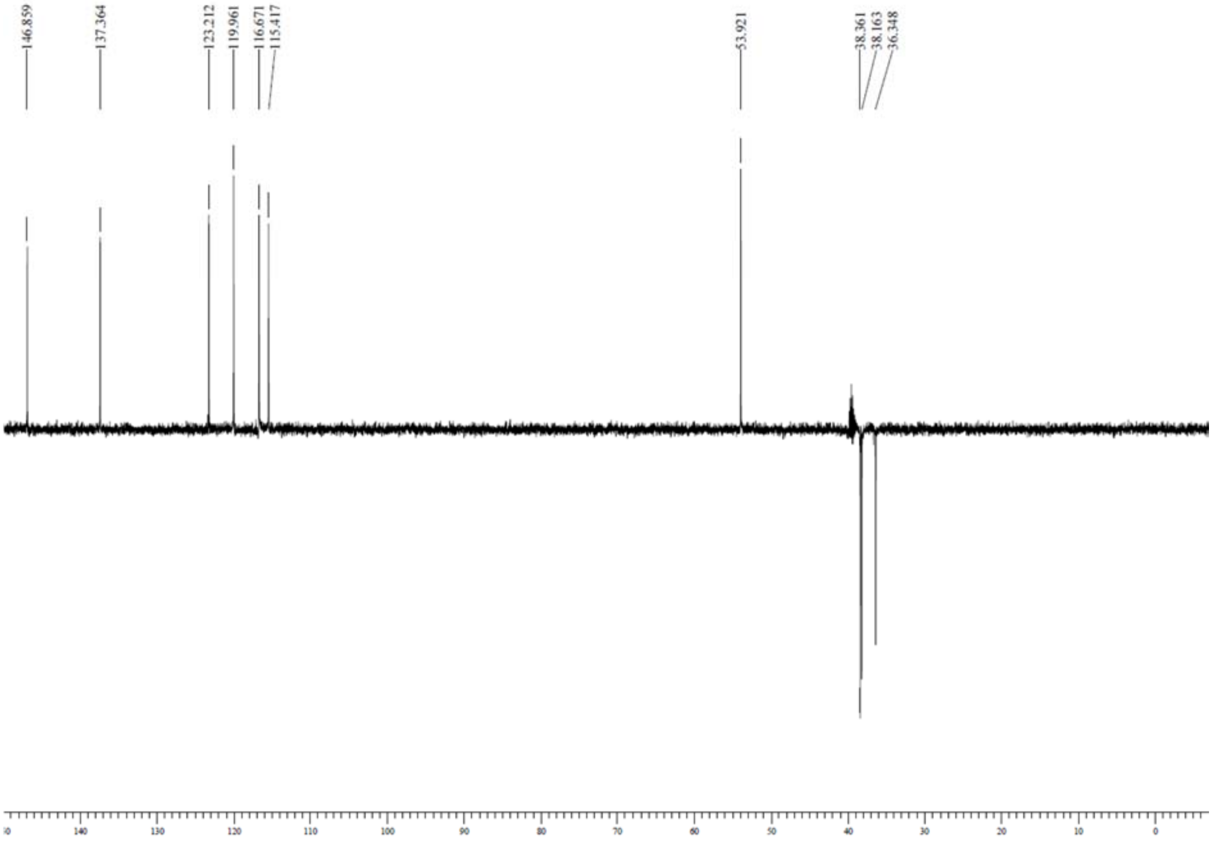

Supplement: Supplementary file 1 [file molecules-22-02076-s001.pdf]
